# Comparison of Growth and Physiological Effects of Soil Moisture Regime on *Plantago maritima* Plants from Geographically Isolated Sites on the Eastern Coast of the Baltic Sea

**DOI:** 10.3390/plants13050633

**Published:** 2024-02-25

**Authors:** Katrīna Anna Ozoliņa, Astra Jēkabsone, Una Andersone-Ozola, Gederts Ievinsh

**Affiliations:** Department of Plant Physiology, Faculty of Biology, University of Latvia, 1 Jelgavas Str., LV-1004 Riga, Latvia; katrina_anna.ozolina@lu.lv (K.A.O.); astra.jekabsone@lu.lv (A.J.); una.andersone-ozola@lu.lv (U.A.-O.)

**Keywords:** chlorophyll, chlorophyll *a* fluorescence, coastal habitats, ion accumulation, osmotic adjustment, *Plantago maritima*, soil flooding, soil water content, waterlogging

## Abstract

The aim of the present study was to evaluate the morphological and physiological responses of *P. maritima* plants from five geographically isolated sites growing in habitats with different conditions to different substrate moisture levels in controlled conditions. Plants were produced from seed and cultivated in a greenhouse at four relatively constant soil moisture regimes: at 25, 50, and 75% soil water content and in soil flooded 3 cm above the surface (80% F). The two morphological traits that varied most strikingly among *P. maritima* accessions were the number of flower stalks and the number of leaves. Only plants from two accessions uniformly produced generative structures, and allocation to flowering was suppressed by both low moisture and flooding. Optimum shoot biomass accumulation for all accessions was at 50 and 75% soil moisture. The Performance Index Total was the most sensitive among the measured photosynthesis-related parameters, and it tended to decrease with an increase in soil water content for all *P. maritima* accessions. The initial hypothesis—that plants from relatively dry habitats will have a higher tolerance against low soil water levels, but plants from relatively wet habitats will have a higher tolerance against waterlogged or flooded soil—was not proven. The existence of three ecotypes of *P. maritima* within the five accessions from geographically isolated subpopulations on the eastern coast of the Baltic Sea at the level of morphological responses to soil water content can be proposed. *P. maritima* plants can be characterized as extremely tolerant to soil waterlogging and highly tolerant to soil flooding and low soil water content.

## 1. Introduction

The physiological and molecular mechanisms of the environmental resilience of wild plants have gained considerable interest for a prolonged period of time [1]. In addition to solving purely natural conservation problems, the assessment of plant features associated with existence in highly heterogeneous conditions, such as ones in coastal habitats, can provide an understanding of plant adaptation mechanisms, which can be useful in various practical developments in the creation of new crop varieties resistant to environmental extremes [2,3,4].

Several environmental factors are important for shaping characteristic vegetation in coastal habitats [5,6,7]. In addition to heterogeneous salinity, soil moisture is another factor that strongly affects the growth and performance of plants native to coastal habitats. The sea coast is a very contrasting place with respect to moisture availability, which is reflected in the different plant adaptations required. Plants in dune habitats usually have distinctly xeromorphic features, allowing them to withstand prolonged periods of water shortage. On the other hand, plants in coastal wetlands are frequently subjected to inundation, and they have properties to tolerate a lack of rootzone oxygen. Also, plants on different types of beaches can exhibit episodes of both drought and flooding. In the non-tidal conditions of the seashore meadows and beaches of the Baltic Sea, flooding can be caused either by seawater as a result of wave activity and changes in sea level or by freshwater due to prolonged rain [8].

Some predominantly coastal-specific plant species occupy a relatively wide range of habitats with different sets of environmental conditions, possibly exhibiting a high degree of phenotypic plasticity. *Plantago maritima* L. is one such species, being found in various salt-affected coastal habitats. *P. maritima* is a self-incompatible, wind-pollinated perennial hemicryptophyte, flowering multiple times during its life span [9]. It is not listed as being a clonal species [10]; however, incidents of clonal expansion have been found both in the field [11] and in controlled conditions [12].

With respect to the EUNIS habitat classifications system, *P. maritima* is a constant species of three coastal habitats, i.e., “Baltic coastal meadow” (MA232), “Atlantic and Baltic rocky sea cliff and shore” (N31), and “Atlantic and Baltic soft sea cliff” (N34) [13]. In general, the species can also be found in other coastal habitats, including salt marshes, mud flats, sea dikes, etc. [14]. According to the ecological indicator values for Sweden, *P. maritima* has been found on mesic–moist soils (indicator value 3 out of 12) and is “competitive only under moderate–high salinity” (indicator value 4 out of 5) [15]. However, according to the indicator list for Great Britain, *P. maritima* is characterized as a “dampness indicator, mainly on constantly moist or damp, but not on wet soils” (indicator value 7 out of 12) and the “species most common in coastal sites but regularly present in freshwater or on non-saline soils inland” (indicator value 3 out of 9) [16].

The salinity responses of *P. maritima* have been widely studied, and it was shown that salt accumulation in shoots [17], the synthesis of organic osmolytes [18], and the induction of the enzymatic antioxidative system [19] are important constituents of the relatively high salinity tolerance of the species. However, given the absence of any growth stimulation due to increased salinity, *P. maritima* is designated as a facultative halophyte [20].

So far, differences in the life characteristics of *P. maritima* populations growing in different conditions have only been seldom assessed. Thus, two salt marsh populations in the Netherlands were compared in a study, and it appeared that the one from the lower zones subjected to tidal flooding and grazing by cattle was long-lived and propagated mainly vegetatively, but the one from the upper zones with low flooding intensity was short-lived and propagated via seeds [11]. These differences appear to be caused by phenotypic plasticity as the result of differences in biotic and abiotic factors. It was concluded that the vegetative spread seen as the formation of daughter rosettes was due to trampling by cattle, and this was confirmed in further greenhouse experiments. Another early study of *P. maritima* in the natural conditions of a seashore meadow along a distributional gradient confirmed that the species, indeed, has high phenotypic plasticity due to differences in salinity, flooding, and light [21]. So far, no conclusive evidence has been obtained on the existence of specific ecotypes of *P. maritima* as a result of local genetic adaptation.

Only a limited amount of information can be found on the responses of *P. maritima* plants to increased soil water content and/or flooding with water. Thus, the waterlogging of *P. maritima* plants from a tidal salt marsh cultivated in salt marsh soil does not result in significant changes in growth irrespective of salinity regime, but the number of dead leaves increases only because of waterlogging in non-saline conditions [22]. Similarly, the growth of *P. maritima* plants is not negatively affected by soil waterlogging or submergence, but the number of dead and severely affected leaves increases by 35 and 50%, respectively [23]. In addition, submergence has a clearly negative effect on the flowering and generative reproduction of *P. maritima* plants [24]. Thus, results from ecological and ecophysiological studies confirm that *P. maritima* is well adapted to high soil water content and even partial submergence, which is in contrast with the reported values of ecological indicators, especially in the Baltic region [15,16]. In addition, the optimum soil moisture regime of *P. maritima* plants in controlled conditions has not been established.

The osmotic protection of the cellular environment is an important feature of coastal plants both in conditions of increased soil salinity and in water shortage situations [5]. It has been argued that halophytic species mostly accumulate inorganic ions (Na^+^ and Cl^−^) to maintain osmotic balance in salinity conditions [25]. However, the accumulation of K^+^ is associated with tolerance to xeric conditions [26]. In particular, the vacuolar pool of K^+^ is an important constituent of osmotic regulation for plants in non-saline conditions [27]. In addition, osmotically active organic compounds (compatible solutes) are important constituents of the water maintenance system in plant tissues exposed to low-water-potential media [28].

In conditions of suboptimal soil water supplies, plant growth is diminished, but the functional reasons for that can differ in the case of low soil moisture (drought) and excessive soil moisture (soil waterlogging). During soil water shortage, the interruption of water flow and decreased turgor pressure in tissues result in negative consequences for cell division and elongation [29,30,31]. Soil waterlogging results in decreased oxygen content, further leading to rootzone hypoxia, the suppression of root respiration, decreased mineral nutrition and photosynthesis, and eventual plant dieback [32]. In addition, soil flooding conditions leading to partial or full plant submergence, the hypoxia of aboveground parts, and post-hypoxia reoxygenation result in the death of plant parts or the whole plant [33]. Endogenous oxidative stress is an inevitable component of functional deterioration in all situations of critically inadequate water provision [31,34,35].

Besides changes in growth, the physiological status of plants is an important indication of plant adaptive success in particular conditions [36]. Usually, photosynthesis-related traits are used as indicators for plant functional status, as photosynthesis is relatively more sensitive to changes in environmental conditions in comparison with growth-related indices [37]. In particular, chlorophyll *a* fluorescence parameters have been used to assess plant tolerance to both drought [38] and flooding [39].

The aim of the present study was to evaluate the morphological and physiological responses of *P. maritima* plants from several geographically isolated sites growing in habitats with different conditions to different substrate moisture levels in controlled conditions. It was hypothesized that plants from relatively dry habitats (such as dry dune grasslands) would have a higher tolerance against low soil water levels, but plants from relatively wet habitats (such as flood-prone grasslands or beaches) would have a higher tolerance to waterlogged or flooded soil. Special emphasis was placed on the possible existence of ecotypes of *P. maritima* in isolated subpopulations in contrast to the phenotypic plasticity of the species.

## 2. Materials and Methods

### 2.1. Plant Material and Seedling Establishment

**Figure 1:**
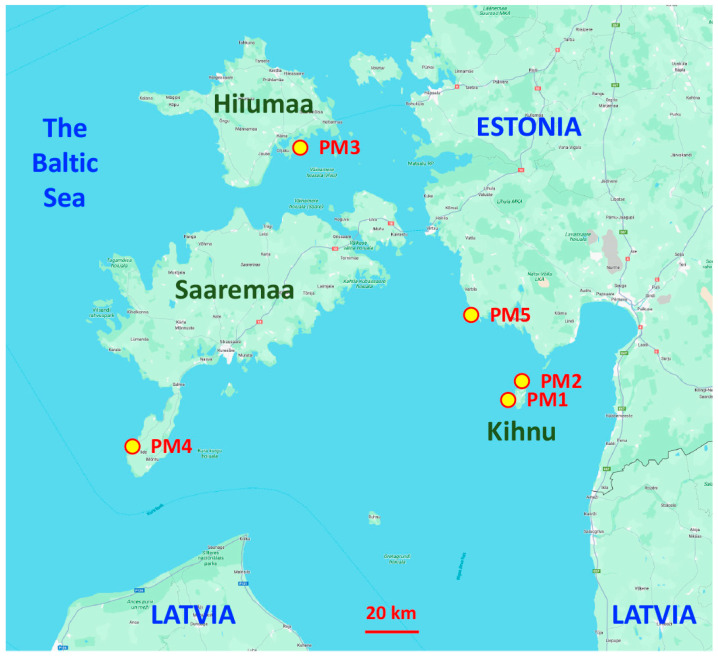
Map of the eastern Baltic Sea region indicating sites for collection of different accessions of *Plantago maritima* in Estonia. Kihnu, Hiiumaa, and Saaremaa are Estonian islands.

Seed material from different geographically isolated subpopulations (accessions) of *P. maritima* was collected in the territory of Estonia in 2022 (Table 1, Figure 1). Sites with *P. maritima* were selected with the idea to include different habitats in terms of environmental conditions (Figure S1). Two sites were located on the island of Kihnu and had contrasting soil moisture conditions (dry sandy dune grassland vs. wet salt-affected meadow). Distance by the coastline between these two sites was about 5 km. In addition to subpopulations of *P. maritima* from the islands of Saaremaa and Hiiumaa, one more accession was obtained from the mainland coastal part of Estonia. Seeds were dried in laboratory conditions for four weeks and then stored at 4 °C until use.

The experiment was performed in the winter season. Seeds were germinated in 1 L tissue culture containers filled with autoclaved commercial garden soil (Biolan, Eura, Finland) [40] mixed with deionized sterile water and placed in a plant growth cabinet, MLR-352H (Sanyo Electric, Osaka, Japan), with a photoperiod of 16 h (photon flux density of photosynthetically active radiation, 40 μmol m^−2^ s^−1^) and day/night temperatures of 20/15 °C. After the formation of the third true leaf, the seedlings were individually transplanted into 220 mL plastic containers containing a mixture of heat-treated (60 °C, 24 h) garden soil (Biolan, Eura, Finland) and quartz sand (Saulkalne S, Saulkalne, Latvia), 4:1 (*v*/*v*). Seedlings were placed in 48 L lid-closed plastic boxes, laid out in a greenhouse, and gradually adapted to greenhouse conditions. An automated experimental greenhouse (HortiMaX, Maasdijk, the Netherlands) with supplemented light provided by Master SON-TPIA Green Power CG T 400W (Philips, Amsterdam, The Netherlands) and Powerstar HQI-BT 400 W/D PRO (Osram, Munich, Germany) lamps (photon flux density of photosynthetically active radiation, 380 μmol m^−2^ s^−1^ at the plant level) with a 16 h photoperiod was used for plant cultivation. The day/night temperature was 23/16 °C, and the relative air humidity was maintained at 60 to 70%.

After seven weeks, plants were individually transplanted into 1.3 L plastic containers with a mixture of garden soil (Biolan, Eura, Finland) and quartz sand (Saulkalne S, Saulkalne, Latvia), 4:1 (*v*/*v*). Plants were watered with deionized water when necessary to maintain substrate water content of 50–60% and measured with an HH2 moisture meter equipped with a WET-2 sensor (Delta-T Devices, Burwell, UK). Individual containers were randomly placed on a greenhouse bench and repositioned once a week. One week after additional acclimatization in the greenhouse, the treatments were started.

### 2.2. Treatments

For each of the five *P. maritima* accessions, four water content regimes were maintained, with five individual plants as replicates. For 25 and 50% treatments, soil water content was measured daily in all containers with an HH2 moisture meter at the four sides of the container. The substrate was supplemented with the necessary amount of deionized water according to a previously performed calibration so that the water content was within 20–30% or 45–55% for the 25 and 50% treatments, respectively. For the 75% treatment, which corresponded to the substrate waterlogging, containers with plants were placed inside 1.5 L plastic containers where 0.5 L of deionized water was maintained. This resulted in a relatively constant substrate water content of 75%. Flooding treatment was performed by placing containers with *P. maritima* plants inside a 4 L plastic container and maintaining a water level at least 3 cm above the soil surface. This resulted in a relatively constant substrate water content of 80%. All treatments lasted for three full weeks, but after that, the flooding treatment was terminated, and the respective plants were maintained in a waterlogged state. Immediately after that, the plants were fertilized with Yara Tera Kristalon Red and Yara Tera Calcinit fertilizers (Yara International, Oslo, Norway). A stock solution was prepared for each fertilizer (100 g L^−1^), and the working solution contained 25 mL of each per 10 L of deionized water, used at a rate of 100 mL per container. The experiment continued for another three weeks.

### 2.3. Plant Harvest and Measurements

One week prior to termination of the experiment, leaf chlorophyll concentration and chlorophyll a fluorescence were measured as described previously using a chlorophyll meter CCM300 (Opti-Sciences, Hudson, NH, USA) and a Handy PEA fluorometer (Hansatech Instruments, King’s Lynn, UK), respectively [40]. For each individual plant, two independent measurements were performed, with 10 measurements per accession per treatment. A fluorescence data analysis was performed using the PEA Plus software (v. 3.11, Hansatech Instruments, King’s Lynn, UK). A number of parameters derived from the fast fluorescence induction curve were used [41,42]. F_v_/F_m_, calculated as (F_m_ − F_0_)/F_m_, represents the maximum quantum efficiency of photosystem II, indicating the probability that a trapped photon will perform a further photochemical energy transfer, and is used as a stress indicator. F_v_/F_0_, calculated as (F_m_ − F_0_)/F_0_, reflects the instant photochemical activity on the donor side of photosystem II. Performance Index Total is used as a relative indication of plant vitality and includes information on the status of both photosystem II and photosystem I, in addition to characterizing the electron flow between the two systems, which is on an absorption basis.

At termination, plants were individually separated into different parts according to morphological traits (roots, old leaves, middle leaves, new leaves, flower stalks, and flowers (both flowers and fruits)). Inflorescences were counted, and the height of flower stalks was measured. All plant material was weighed separately before and after drying in an oven at 60 °C for 72 h. Tissue water content was calculated as g of H_2_O per g of dry mass.

Electrical conductivity, concentrations of Na^+^ and K^+^, and osmotic values in water extracts of dry plant material were measured as described previously [43]. Electrical conductivity was measured using a LAQUAtwin B-771 conductivity meter, Na^+^ concentration was measured using a LAQUAtwin B-722 compact meter, and K^+^ concentration was measured using a LAQUAtwin B-731 compact meter (Horiba, Kyoto, Japan). Osmotic activity in tissue extracts was measured by a freezing point osmometer Osmomat 3000 Basic (Gonotec Meß- und Regeltechnik, Berlin, Germany). Using a standard curve for different concentrations of NaCl and KCl, the osmotic value caused by the total concentration of Na^+^ and K^+^ was calculated according to the actual Na^+^ and K^+^ concentrations of each sample extract. For each sample, the difference between the total osmotic value and the osmotic value due to Na^+^, K^+^, and Cl^−^ ions was calculated and designated as the “non-ionic osmotic value”, which showed the osmotic effect of other osmotically active ions (besides Na^+^, K^+^, and Cl^−^) or non-ionic compounds. Six tissue samples were independently measured for each accession/soil water content combination.

### 2.4. Data Analysis

All data were entered into MS Excel 365 (v. 16.81, Microsoft Corporation, Redmond, WA, USA). Data were analyzed using JASP (v. 0.17.2.1, University of Amsterdam, Amsterdam, the Netherlands). ANOVA analysis with a Tukey HSD test was performed at *p* < 0.05. Graphs were generated using RStudio (v. 4.2.2, R Foundation for Statistical Computing, Vienna, Austria). Principal component analysis and heat map generation were performed using ClustVis (http://biit.cs.ut.ee/clustvis/, accessed on 5 January 2024), a freely available web program [44]. Hierarchical clusters were generated using the average linkage method with correlation distance.

## 3. Results

### 3.1. Morphological Parameters

**Figure 2:**
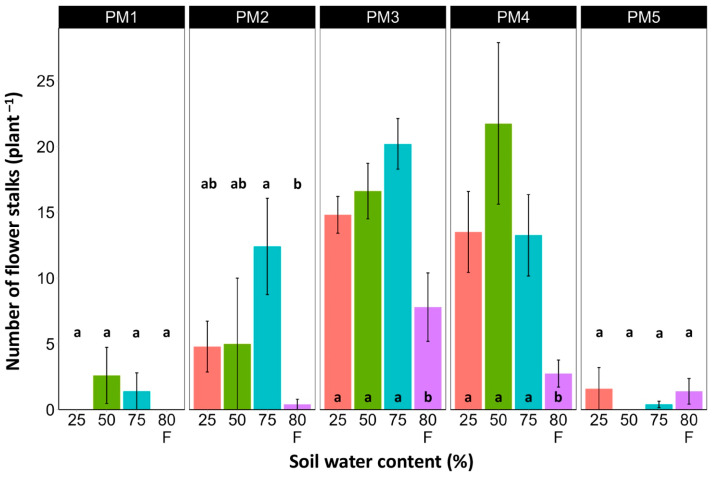
Effects of soil moisture and flooding (F) on the number of flower stalks of *Plantago maritima* plants from different accessions. Data are means ± SE from five replicates. Different letters indicate statistically significant differences according to the Tukey HSD test (*p* < 0.05) within the particular accession.

**Figure 3:**
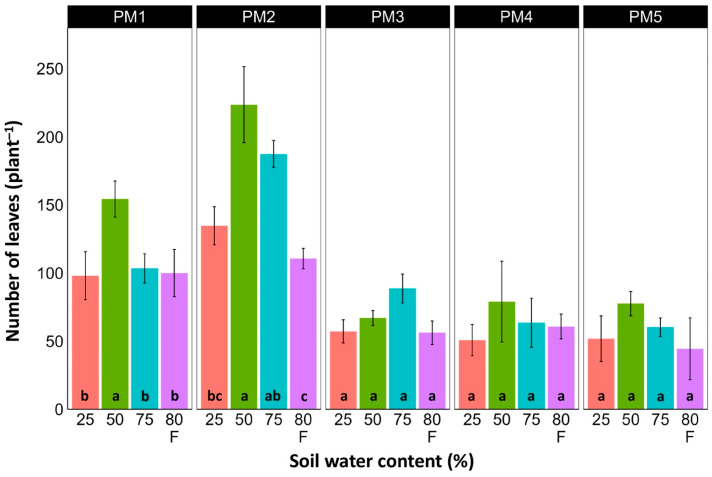
Effects of soil moisture and flooding (F) on the number of leaves of *Plantago maritima* plants from different accessions. Data are means ± SE from five replicates. Different letters indicate statistically significant differences according to the Tukey HSD test (*p* < 0.05) within the particular accession.

At the end of the experiment, *P. maritima* plants showed morphological differences between various accessions, as well as because of variations in soil moisture (Figures S2–S6). The two morphological traits that varied most strikingly among *P. maritima* accessions were the number of flower stalks and the number of leaves. Individuals of accessions PM1 and PM2 showed a low and variable ability to form generative organs (Figure 2) and had a relatively high number of leaves (Figure 3). Plants of accessions PM3 and PM4 formed many flower stalks and had a lower number of leaves in comparison with PM1 and PM2. However, PM5 plants had both a very low capacity for generative reproduction, as well as a low number of leaves. Given the clear genotype-related differences between *P. maritima* accessions, the effect of soil moisture on these two parameters also varied. For PM3 and PM4, plants in flooded conditions had a significantly decreased number of flower stalks, and the same was evident for PM2 (Figure 2). The highest number of leaves developed for PM1, PM2, PM4, and PM5 plants cultivated at 50% soil moisture, but for PM3 plants, this occurred at 75% soil moisture (Figure 3). However, because of the relatively large individual variability in leaf number, these effects for PM3, PM4, and PM5 plants were not statistically significant.

Other parameters of PM3 and PM4 related to generative reproduction were more sensitive to differences in soil moisture. Thus, biomass allocated to flower stalks was low at 25% soil moisture and increased for plants at 50 and 75% moisture, but the effect was statistically significant only for PM3 plants (Table 2). In the case of PM4, a significant decrease in flower stalk biomass was observed in flooded plants. Similarly, the biomass of flowers and fruits was low at 25% moisture, increased at 50 and 75% moisture, and decreased again for flooded plants (Table 2). However, for PM4 plants, this effect was significant only for flooded plants.

**Figure 4:**
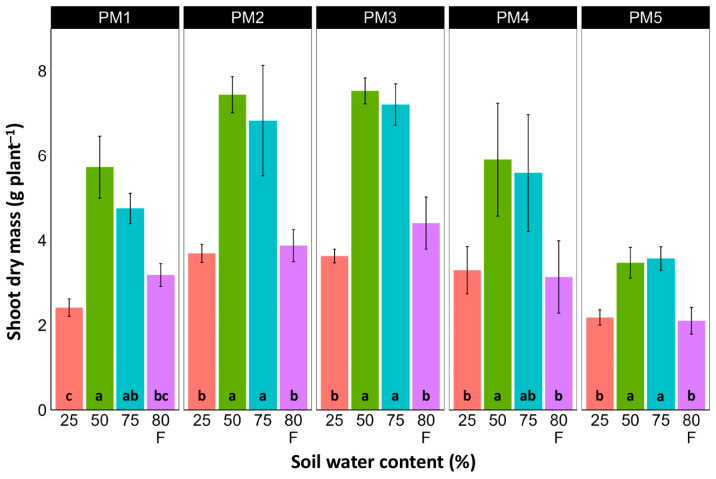
Effects of soil moisture and flooding (F) on the dry mass of shoots of *Plantago maritima* plants from different accessions. Data are means ± SE from five replicates. Different letters indicate statistically significant differences according to the Tukey HSD test (*p* < 0.05) within the particular accession.

**Figure 5:**
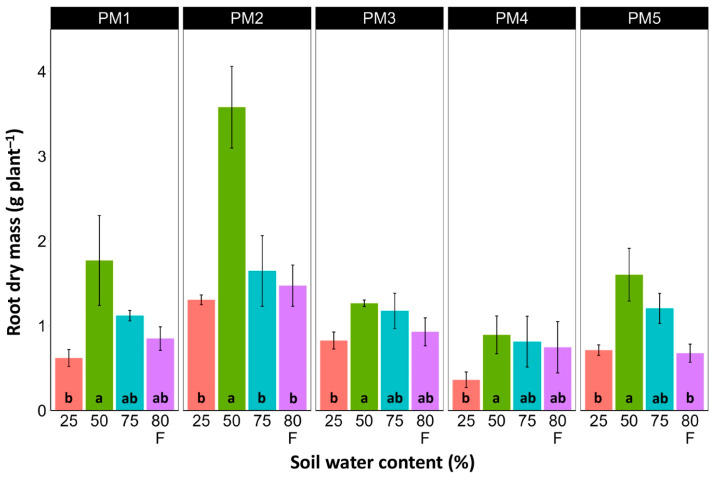
Effects of soil moisture and flooding (F) on the dry mass of roots of *Plantago maritima* plants from different accessions. Data are means ± SE from five replicates. Different letters indicate statistically significant differences according to the Tukey HSD test (*p* < 0.05) within the particular accession.

In spite of different biomass investment strategies between plants from the PM1, PM2 (toward leaf development and growth), PM3, and PM4 (toward generative reproduction) accessions, the total shoot biomass at the optimum soil moisture (50 and 75%) of these plants was similar (Figure 4). However, PM5 plants had lower biomass values. For roots, the highest biomass for all accessions was in 50% soil moisture conditions, but these differences were statistically significant in all other moisture regimes only for PM2 plants (Figure 5). In addition, for all accessions, root biomass was significantly lower for plants cultivated at 25% soil moisture in comparison with that at 50% moisture. Moreover, a significant decrease in root biomass was also evident for flooded PM5 plants.

**Figure 6:**
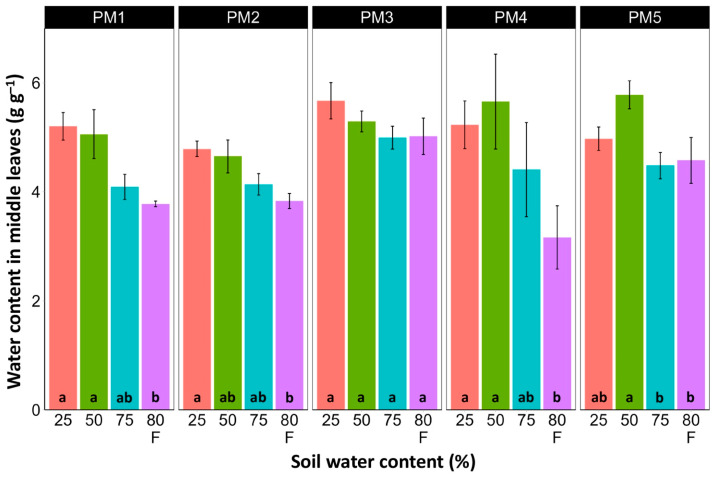
Effects of soil moisture and flooding (F) on water content in middle leaves of *Plantago maritima* plants from different accessions. Data are means ± SE from five replicates. Different letters indicate statistically significant differences according to the Tukey HSD test (*p* < 0.05) within the particular accession.

**Figure 7:**
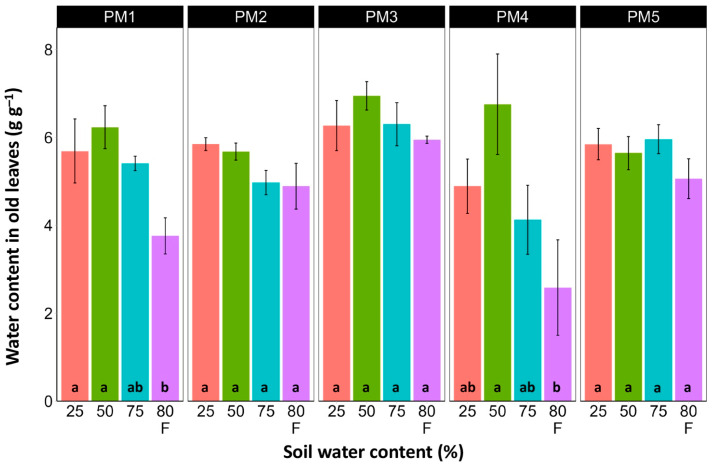
Effects of soil moisture and flooding (F) on water content in old leaves of *Plantago maritima* plants from different accessions. Data are means ± SE from five replicates. Different letters indicate statistically significant differences according to the Tukey HSD test (*p* < 0.05) within the particular accession.

Water content was estimated in the middle (Figure 6) and old leaves (Figure 7) of *P. maritima* plants cultivated at different soil moisture regimes in order to monitor any preliminary signs of leaf senescence. While plants at 25, 50, and 75% soil moisture showed no significant changes in leaf water content, flooding resulted in a significant decrease in this parameter for plants of all accessions, except PM3 in the case of middle leaves, but for old leaves, this effect was significant only for PM1 and PM4 plants.

**Figure 8:**
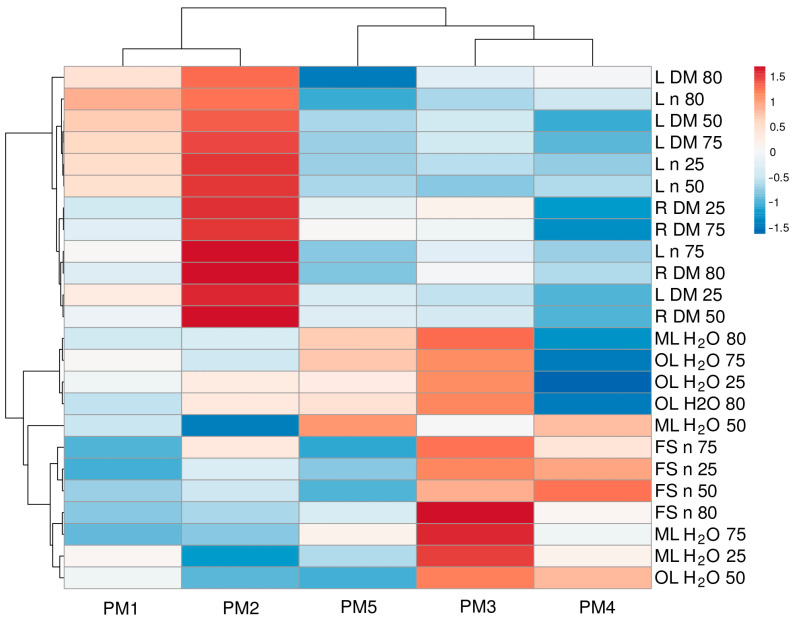
Generated heat map and cluster analysis of the effect of soil moisture and flooding on morphological parameters in *Plantago maritima* plants from different accessions. Hierarchical clusters were generated via the average linkage method with correlation distance; color scale shows relative intensity of normalized parameter values. DM, dry mass; FS, flower stalks; H_2_O, water content; L, leaves; ML, middle leaves; n, number; OL, old leaves; R, roots. The numbers 25, 50, 75, and 80 indicate soil water content (%).

To characterize the similarity between genotypes in relation to morphological parameters and their changes under the influence of changing soil moisture, multivariate analysis was performed (Figure 8). Accessions PM1 and PM2 were the most similar, and another pair with a somewhat lower degree of similarity was formed by PM3 and PM4. However, PM5 formed a separate cluster. It is also interesting to note that, among morphological traits, those related to leaf number and biomass, as well as root biomass, clustered together. Another more structured cluster was formed by traits such as water content and the number of flower stalks.

### 3.2. Accumulation of Ions and Osmotic Activity

For the characterization of total soluble ion content, electrical conductivity in leaf extracts was measured in the middle leaves of *P. maritima* plants cultivated at different soil moisture levels (Table 3). In general, the highest level of electrical conductivity was in plants cultivated at 25% soil moisture, which decreased with increasing soil moisture, with the lowest level in flooded plants. This effect was statistically significant already at 50% soil moisture for PM1 and PM3 plants but only for flooded plants of accessions PM2, PM3, and PM4. The concentration of Na^+^ also showed a tendency to decrease with increasing soil water content, but statistically significant differences were evident for PM1, PM2, and PM4 plants only in flooded conditions and for PM3 plants also at 75% soil moisture (Table 3). The concentration of K^+^ significantly decreased for PM2 in leaves of plants cultivated at 75% and flooded plants, as well as for PM3 at 50% and flooded PM5 plants, in comparison with the values at 25% (Table 3). The total osmotic activity was relatively stable, especially for PM3 and PM5 plants and other accessions at low or moderate soil moisture (PM1), or decreased significantly only for flooded plants (PM2, PM4). The activity of non-ionic osmolytes did not change with differences in soil water content in PM1 and PM5, but it increased at 50 and 75% (PM2) or only 50% (PM3), while a significant decrease was evident for flooded PM4 plants.

The variation in these parameters in roots between various accessions under the effect of differences in soil moisture was less pronounced (Table S1). For electrical conductivity, a statistically significant difference was observed between plants at 50 and 70% soil moisture only for PM3. For Na^+^ concentration, PM2 plants at 75% and PM4 plants at 50% had values significantly higher than those of the rest of the moisture levels, and Na^+^ at 25% was higher than at 50 and 75% for PM5. The only statistically significant differences for root K^+^ concentration were between 50 and 75% for PM2 and 25, 50%, 75, and 80% for PM3. More differences were evident for total osmotic activity in roots, where there was a tendency for plants at low or moderate soil water levels (25 and 50%) to have lower values in comparison with those of flooded plants at the high water level. There were no statistically significant differences for root non-ionic osmotic activity except between plants at 50 and 75% soil moisture for PM3.

Because of the rather variable responses in the ion accumulation and osmotic activity of different *P. maritima* accessions cultivated at different soil moisture percentages, the cluster analysis revealed that each accession had a rather unique response distribution picture, but the highest similarity was between accessions PM4 and PM5 (Figure S7).

### 3.3. Photosynthesis-Related Parameters

**Figure 9:**
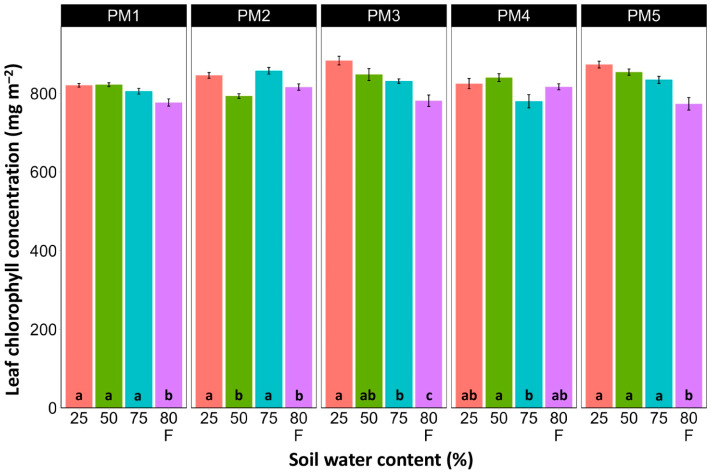
Effects of soil moisture and flooding (F) on chlorophyll concentration in middle leaves of *Plantago maritima* plants from different accessions. Data are means ± SE from five replicates, with two measurements each. Different letters indicate statistically significant differences according to the Tukey HSD test (*p* < 0.05) within the particular accession.

**Figure 10:**
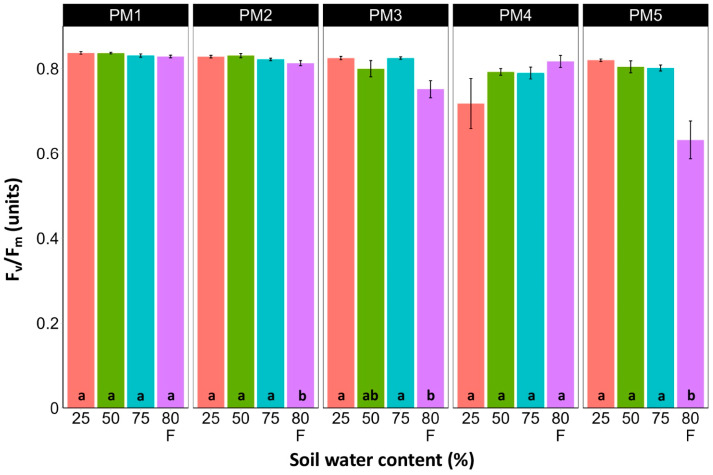
Effects of soil moisture and flooding (F) on chlorophyll *a* fluorescence parameter F_v_/F_m_ in middle leaves of *Plantago maritima* plants from different accessions. Data are means ± SE from five replicates, with two measurements each. Different letters indicate statistically significant differences according to the Tukey HSD test (*p* < 0.05) within the particular accession.

**Figure 11:**
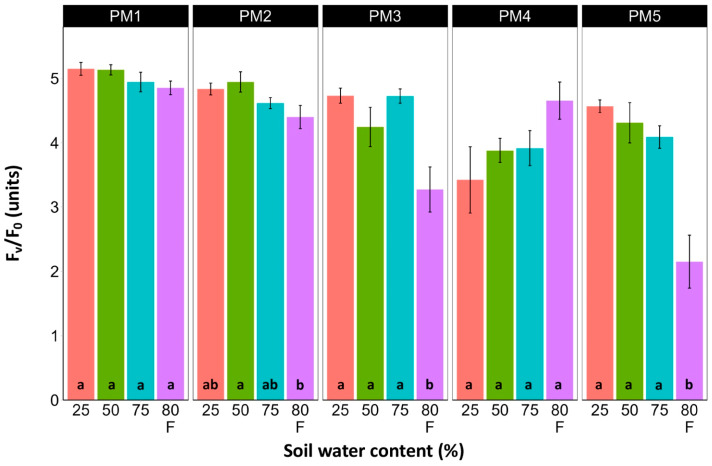
Effects of soil moisture and flooding (F) on chlorophyll *a* fluorescence parameter F_v_/F_0_ in middle leaves of *Plantago maritima* plants from different accessions. Data are means ± SE from five replicates, with two measurements each. Different letters indicate statistically significant differences according to the Tukey HSD test (*p* < 0.05) within the particular accession.

**Figure 12:**
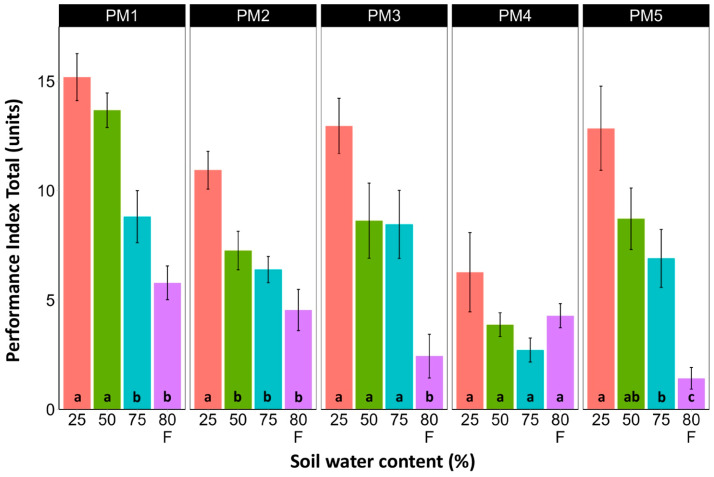
Effects of soil moisture and flooding (F) on chlorophyll *a* fluorescence parameter Performance Index Total in middle leaves of *Plantago maritima* plants from different accessions. Data are means ± SE from five replicates, with two measurements each. Different letters indicate statistically significant differences according to the Tukey HSD test (*p* < 0.05) within the particular accession.

Leaf chlorophyll concentration in *P. maritima* accessions PM3 and PM5 showed a tendency to decrease with increasing soil water content, but a statistically significant effect was evident only at 75% and with flooding (PM3) or flooding alone (PM5) (Figure 9). In addition, accessions PM1 and PM2 also had lower chlorophyll concentrations during flooding in comparison with those at 25% soil moisture. The chlorophyll *a* fluorescence parameter F_v_/F_m_ was relatively insensitive to changes in soil water content, but significantly lower values of the parameter were seen in all accessions except PM4 for flooded plants (Figure 10). The character of changes in another fluorescence parameter, F_v_/F_0_, was similar to that of F_v_/F_m_, but to a greater extent (Figure 11). Significantly lower values of F_v_/F_0_ for flooded plants were evident for accessions PM2, PM3, and PM4. The Performance Index Total was the most sensitive among the measured photosynthesis-related parameters, and it tended to decrease with increases in soil water content for all *P. maritima* accessions, but the differences were not always statistically significant (Figure 12).

Multivariate analysis revealed a relatively close similarity between PM3 and PM5 at the level of photosynthesis-related parameter responses (Figure S8). At the association level of the measured parameters, leaf chlorophyll concentration showed a close relationship in plants at all soil moisture levels except flooding. On the other hand, all parameters for flooded plants are also clustered together.

## 4. Discussion

Usually, the effects of either drought episodes or high soil moisture and flooding on plants are studied separately in the context of plant moisture regimes [45]. Very often, changes in soil moisture are not precisely monitored, and because of variations in other factors, the results obtained in such experiments are difficult to generalize. The present results show that differences in plant growth and the physiological state of plants due to stable differences in soil moisture from moderate insufficiency to waterlogging can be viewed as a continuous phenomenon. However, flooding-dependent plant submergence results in qualitatively distinct reactions, both at the signal perception and morphophysiological response levels [35,46]. It needs to be noted, however, that soil moisture in coastal habitats is extremely heterogeneous both in space and time [5]. Therefore, plants can be exposed to drought/excess moisture and flooding episodes of different durations and intensities, which can have different effects.

In general, *P. maritima* plants were extremely tolerant to soil waterlogging and highly tolerant to both soil flooding and low water content. The growth and development of plants were significantly reduced both in low moisture and flooded conditions, but no visual signs of disturbance were evident. Another characteristic species of salinity- and flooding-affected coastal habitats, *Trifolium fragiferum*, showed signs of metabolic disturbance even after several weeks of flooding, such as leaf chlorosis, accompanied by a severe reduction in plant growth [47]. *Saussurea esthonica* plants, typical for calcareous fens, had maximum growth and development in waterlogged conditions but showed symptoms of metabolic disorder in the form of purple regions between main leaf veins (after four weeks of waterlogging) and chlorotic lesions (after six weeks of waterlogging) [48].

There was no relationship between potential differences in daily water regime at the growth sites and morphological responses to experimentally induced water changes in the different *P. maritima* accessions. Thus, the initial hypothesis was not confirmed. All accessions had the highest shoot biomass at 50 and 75% soil moisture, showing exceptional tolerance to soil waterlogging (Figure 4). The relative decrease in biomass in low soil moisture (25%) or flooded conditions (80% F) was similar between both regimes and for all accessions. However, significantly lower water content in the older leaves of previously flooded plants of PM1 and PM4 indicated that, over a longer period of time, the effects of the treatment could manifest as the dieback of the older leaves (Figure 7). This was in part confirmed by the significant decrease in leaf chlorophyll concentrations in flooded plants (Figure 9). In addition, chlorophyll *a* fluorescence parameter F_v_/F_m_, an indicator of long-term unfavorable environmental influence [49], significantly decreased only in plants in flooded conditions for PM2, PM 3, and PM 5 (Figure 10), and for PM5, it was below 0.7, possibly showing the existence of negative metabolic changes. The reduced water content of the middle leaves in flooded plants (Figure 6) is more difficult to explain but could also be related to adverse metabolic changes. In comparative experiments with several plant species, it has been proven that chlorophyll fluorescence measurements can be used to distinguish flooding-sensitive plants from flooding-tolerant plants [39], indicating that the ability to sustain photochemical activity during flooding is an important adaptive mechanism. However, even native wetland species with a high tolerance to submergence show a decrease in photosynthesis-related parameters in parallel to growth inhibition with an increase in flooding depth [50].

Initial experiments looked for *P. maritima* ecotypes within the same geographic population among plants that grow in different areas of salt marshes [11] or along a distributional gradient in seashore meadows [21] and show distinguishing morphological characteristics. However, when cultivated in identical environmental conditions, *P. maritima* plants from different microenvironments show uniform growth and morphology, confirming the high phenotypic plasticity of the species. In the present study, however, seed material from *P. maritima* plants growing in geographically isolated sites was used. Only PM1 and PM2 plants were from the same island, but they were found to persist in different conditions (dry dune grasslands vs. wet saline grasslands).

One of the most striking morphological differences between the accessions of *P. maritima* was at the level of flower induction (Figure 2). The photoperiodic requirements for flowering in *P. maritima* are not known. However, a 16 h photoperiod has been used to induce flowering in *Plantago lanceolata* [51]. Similarly, an increase in the light period from 12 to 16 h increases the number of flowering *Plantago major* individuals [52]. The present experiment was performed in the winter season, meaning that the length of natural daylight had no effect on the plants, and a 16 h photoperiod with 8 h of darkness was not an appropriate induction factor for flowering for several accessions of *P. maritima*, which probably require even shorter nights. Low light intensity due to dense vegetation prevents flowering in *P. maritima* [21]. However, in natural conditions, flowering is a factor that increases individual mortality, especially during low salinity and decreased flooding frequency and duration [21].

*P. maritima* can be characterized as a salt-accumulating species that controls the total electrolyte content in its leaves in native coastal habitat conditions via changes in Na^+^ concentration against the background of stable and low K^+^ concentrations [53]. Soil waterlogging did not affect Na^+^ and K^+^ concentrations in the shoots of *P. maritima* plants [22]. In the present study, soil moisture affected the accumulation of soluble ions in the leaves of *P. maritima*, but the effect was clearly genotype-specific (Table 3). In general, there was a decrease in the electrical conductivity of tissue extracts with increasing soil moisture, which was associated in part with a decrease in Na^+^ and K^+^ concentrations. No positive relationship between growth at low soil moisture and K^+^ accumulation was found.

Osmotic protection in *P. maritima* in saline conditions has been attributed to the accumulation of both inorganic ions (Na^+^, Cl^−^) [20] and sorbitol [18]. Total osmotic activity in the leaves of *P. maritima* was stable between 25 and 50% soil moisture but decreased significantly for flooded PM1, PM2, and PM4 plants (Table 3). However, the activity of non-ionic osmolytes showed differential responses to soil moisture, as it was higher for PM2 plants at 50 and 75% and PM3 plants at 50% while lower for flooded PM4 plants. Thus, in general, the osmotic adjustment system was not involved in adaptation to various moisture regimes, at least on the dry soil side.

Among the photosynthesis-related indicators of plant physiological status, the Performance Index Total was the most sensitive parameter to changes in soil moisture (Figure 12). However, as the optimum growth of *P. maritima* plants was at 50–75% soil moisture and the highest values of the Performance Index Total were at 25% soil moisture, this indicates that this parameter cannot be used as the sole indicator of the physiological status of plants. A similar discrepancy between fluorescence indicators and biomass production under the influence of various factors, including soil moisture, has also been shown in other studies [48]. From a physiological point of view, it has been argued that photosynthesis and the expansive growth of plants under different water availability situations are independently controlled [30]. As a result, these two processes can be uncoupled even for prolonged periods of time and show different responses to changes in soil water content.

The existence of three morphological ecotypes can be proposed between the studied accessions of *P. maritima*. First, two accessions, PM1 and PM2, from the island of Kihnu showed close association at the level of responses to variation in soil moisture and flooding, irrespective of their native location in habitats with contrasting soil moisture regimes. Thus, the initial hypothesis—that the plants from relatively dry habitats will have higher tolerance against low soil water levels, but the plants from relatively wet habitats will have higher tolerance against waterlogged or flooded soil—was not approved. Second, only plants from accessions PM3 and PM4 showed the full potential of generative reproduction in the conditions of the experiment. Third, PM5 plants stood out with a low number of leaves with low total biomass (similar to those of PM3 and PM4), but the plants did not flower in the conditions of the experiment. Consequently, there is reason to believe that geographically isolated but relatively close subpopulations contain genetically distinct material, possibly resulting from local genetic adaptation or, more likely, from different seed sources during post-glacial colonization. There is no indication of the water-assisted seed dispersal of *P. maritima* or any other *Plantago* species [15], but the close association of *P. maritima* with coastal habitats in the Baltic Sea region, as well as the complete recovery of seed germination after prior exposure to saline water [54,55], means that we cannot discount this possibility. Molecular genetic studies of *P. maritima* accessions from a wider geographical region of the Baltic Sea are necessary to find out the degree of genetic relatedness between isolated subpopulations in order to determine the biological diversity of the species.

## Figures and Tables

**Table 1:** Sites and habitat conditions of *Plantago maritima* accessions in Estonia used in the present study.

Accession	Site	Location	Habitat	Coordinates
PM1	Linaküla	Island of Kihnu	Dry dune grassland	58°07′51.9″ N 23°57′35.8″ E
PM2	Sääre	Island of Kihnu	Wet saline grassland with high plant diversity	58°09′16.4″ N 24°00′13.0″ E
PM3	Kassari	Island of Hiiumaa	Wet saline grazed grassland with low plant diversity	58°48′36.6″ N 22°52′00.2″ E
PM4	Ohesaare	Island of Saaremaa	Dominant *Plantago maritima* stand on pebbly beach	58°00′06.4″ N 22°01′26.3″ E
PM5	Matsi-Sömeri	Pärnu County	Flood-prone stony beach	58°21′02.9″ N 23°44′30.3″ E

**Table 2:** Effects of soil moisture and flooding (F) on average height of flower stalks, dry mass of flower stalks, and dry mass of flowers and fruits of *Plantago maritima* plants of accessions PM3 and PM4.

Soil Moisture	Average Height of Flower Stalk (mm)		Dry Mass of Flower Stalks (g plant^−1^)		Dry Mass of Flowers and Fruits (g plant^−1^)	
	PM3	PM4	PM3	PM4	PM3	PM4
25%	144 ± 16 c	199 ± 18 bc	0.55 ± 0.05 cd	0.71 ± 0.16 bcd	1.15 ± 0.07 c	0.91 ± 0.19 c
50%	242 ± 7 ab	226 ± 17 ab	1.71 ± 0.13 a	1.42 ± 0.28 ab	2.43 ± 0.29 a	1.62 ± 0.28 abc
75%	189 ± 22 bc	221 ± 22 ab	1.33 ± 0.16 ab	1.33 ± 0.07 ab	2.13 ± 0.15 ab	1.34 ± 0.20 bc
80% F	273 ± 10 a	215 ± 5 ab	1.08 ± 0.21 abc	0.22 ± 0.03 d	1.14 ± 0.24 c	0.18 ± 0.01 d

Data are means ± SE from five replicates. Different letters indicate statistically significant differences according to the Tukey HSD test (*p* < 0.05) for the particular parameter.

**Table 3:** Effects of soil moisture and flooding (F) on electrical conductivity, Na^+^ concentration, K^+^ concentration, and osmotic activity in middle leaves of *Plantago maritima* plants from different accessions.

Soil Moisture	PM1	PM2	PM3	PM4	PM5
Electrical conductivity (mS m^−1^ kg^−1^ DM)					
25%	108.6 ± 3.4 a	107.9 ± 3.3 a	106.1 ± 3.3 ab	95.9 ± 6.9 abcd	88.2 ± 1.7 cde
50%	90.5 ± 5.2 bcde	98.1 ± 4.1 abcd	88.3 ± 0.9 cde	92.7 ± 5.1 abcd	100.6 ± 1.8 abc
75%	85.2 ± 4.0 cde	93.4 ± 2.2 abcd	85.2 ± 3.2 cde	91.3 ± 5.5 abcde	81.2 ± 3.8 def
80% F	73.9 ± 1.9 efg	66.2 ± 2.2 fg	73.7 ± 4.0 efg	62.3 ± 3.4 g	63.4 ± 2.6 g
Na^+^ concentration (g kg^−1^ DM)					
25%	5.22 ± 0.21 a	4.03 ± 0.20 abcd	5.23 ± 0.23 a	5.18 ± 0.19 a	3.10 ± 0.33 cde
50%	4.24 ± 0.43 abcd	4.05 ± 0.33 abcd	5.08 ± 0.38 a	4.94 ± 0.75 ab	4.49 ± 0.13 abc
75%	3.90 ± −0.26 abcd	3.16 ± 0.14 cde	3.44 ± 0.28 bcd	4.44 ± 0.36 abc	2.92 ± 0.23 de
80% F	3.07 ± 0.13 cde	2.37 ± 0.07 e	3.28 ± 0.07 e	2.90 ± 0.14 de	2.44 ± 0.09 e
K^+^ concentration (g kg^−1^ DM)					
25%	23.2 ± 1.4 ab	22.1 ± 1.4 ab	18.6 ± 1.4 abcd	22.1 ± 4/5 ab	21.7 ± 0.9 abc
50%	23.3 ± 1.2 a	16.5 ± 0.9 bcdef	9.8 ± 0.8 f	20.1 ± 0.8 abc	24.1 ± 0.8 a
75%	21.3 ± 1.1 abc	15.1 ± 0.6 cdef	12.9 ± 1.8 def	22.8 ± 2.5 ab	20.0 ± 1.1 abc
80% F	18.5 ± 0.6 abcd	11.6 ± 0.7 ef	12.0 ± 0.6 def	17.4 ± 0.6 def	12.5 ± 0.6 def
Osmotic activity (osmol kg^−1^ DM)					
25%	3.25 ± 0.12 a	2.85 ± 0.04 abcdef	3.17 ± 0.09 abc	3.09 ± 0.05 abc	2.84 ± 0.08 bcdef
50%	3.09 ± 0.11 abc	3.04 ± 0.08 abcd	3.07 ± 0.07 abc	3.13 ± 0.11 abc	3.18 ± 0.03 aab
75%	2.82 ± 0.10 bcdef	2.78 ± 0.03 cdef	2.97 ± 0.09 abcdef	3.12 ± 0.05 abc	2.81 ± 0.10 bcdef
80% F	2.65 ± 0.08 defg	2.27 ± 0.05 g	2.99 ± 0.06 abcde	2.61 ± 0.06 efg	2.57 ± 0.07 fg
Non-ionic osmotic activity (osmol kg^−1^ DM)					
25%	1.42 ± 0.06 defg	1.18 ± 0.06 g	1.57 ± 0.09 bcde	1.61 ± 0.06 abcde	1.26 ± 0.08 efg
50%	1.33 ± 0.05 defg	1.63 ± 0.04 abcd	1.90 ± 0.08 a	1.44 ± −0.07	1.38 ± 0.03 defg
75%	1.19 ± 0.05 g	1.52 ± 0.05 cdef	1.78 ± −0.08 abc	1.41 ± 0.08 defg	1.32 ± 0.05 defg
80% F	1.22 ± 0.07 fg	1.24 ± 0.06 fg	1.86 ± 0.04 ab	1.24 ± 0.03 fg	1.49 ± 0.09 cdefg

Data are means ± SE from five replicates. Different letters indicate statistically significant differences according to the Tukey HSD test (*p* < 0.05) for the particular parameter. DM, dry mass.

## Data Availability

All data reported here are available from the authors upon request.

## Supplementary Materials

The following supporting information can be downloaded at https://www.mdpi.com/article/10.3390/plants13050633/s1: Figure S1: Sites with *Plantago maritima* accessions in Estonia. Figure S2: Typical *Plantago maritima* PM1 plants six weeks after the start of the treatment (two weeks after the flooding treatment was terminated). Figure S3: Typical *Plantago maritima* PM2 plants six weeks after the start of the treatment (two weeks after the flooding treatment was terminated). Figure S4: Typical *Plantago maritima* PM3 plants six weeks after the start of the treatment (two weeks after the flooding treatment was terminated). Figure S5: Typical *Plantago maritima* PM4 plants six weeks after the start of the treatment (two weeks after the flooding treatment was terminated). Figure S6: Typical *Plantago maritima* PM5 plants six weeks after the start of the treatment (two weeks after the flooding treatment was terminated). Figure S7: Generated heat map and cluster analysis of the effect of soil moisture and flooding on ion accumulation and osmotic activity in *Plantago maritima* plants from different accessions. Figure S8: Generated heat map and cluster analysis of the effect of soil moisture and flooding on photosynthesis-related parameters in middle leaves of *Plantago maritima* plants from different accessions. Table S1: Effects of soil moisture and flooding (F) on electrical conductivity, Na^+^ concentration, K^+^ concentration, and osmotic activity in roots of *Plantago maritima* plants from different accessions.
