# Supplementary material for: Comparison of Growth and Physiological Effects of Soil Moisture Regime on Plantago maritima Plants from Geographically Isolated Sites on the Eastern Coast of the Baltic Sea
Source: Plants (Basel). 2024 Feb 25;13(5):633. doi: 10.3390/plants13050633 (PMC10934305; doi:10.3390/plants13050633)
Supplement: Supplementary file 1 [file plants-13-00633-s001.zip › Suppl Figures.pdf]

Comparison of Growth and Physiological Effects of Soil Moisture Regime on *Plantago maritima* Plants from Geographically Isolated Sites on the Eastern Coast of the Baltic Sea

Katrīna Anna Ozoliņa, Astra Jēkabsone, Una Andersone-Ozola and Gederts Ievinsh

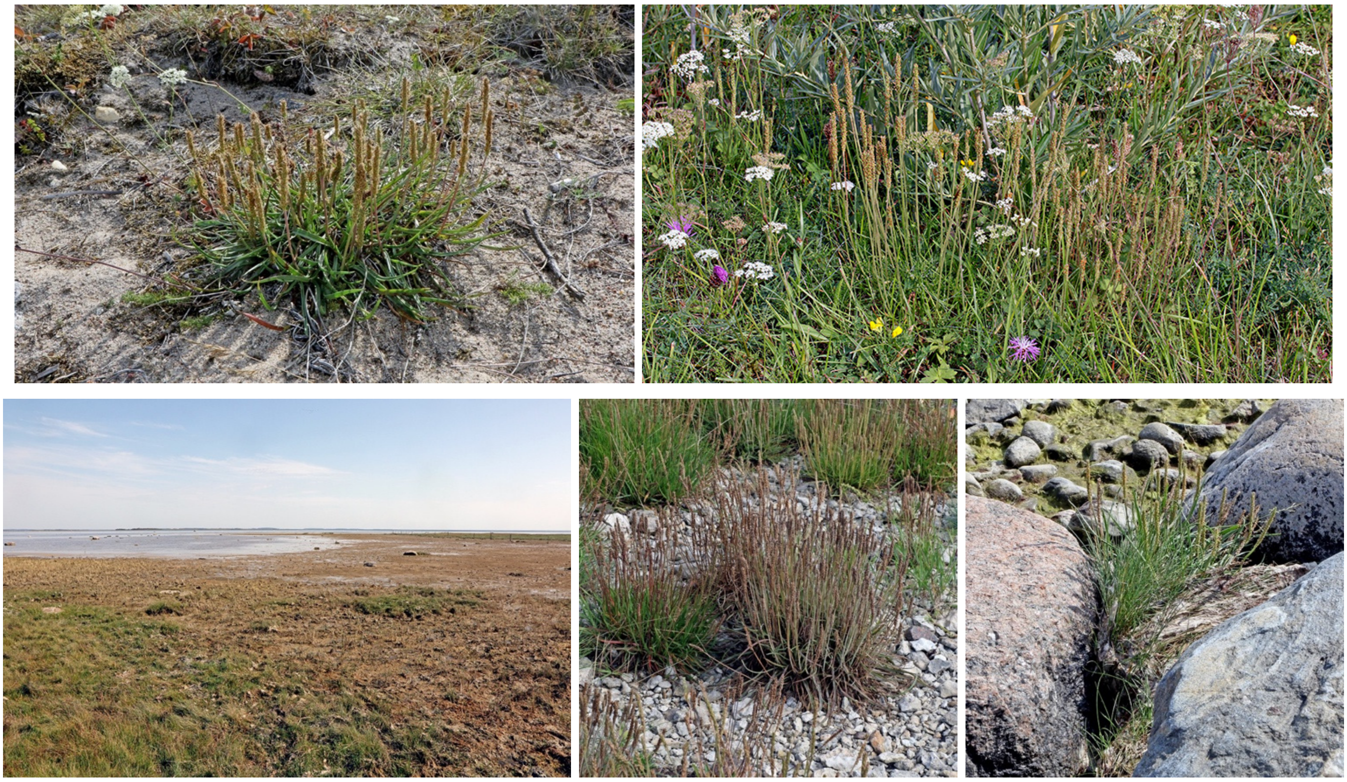

Figure S1. Sites with *Plantago maritima* accessions in Estonia. Top row from left: dry dune grassland (island of Kihnu, PM1), wet saline grassland with high plant diversity (island of Kihnu, PM2). Bottom row from left: wet saline grazed grassland with low plant diversity (island of Hiiumaa, PM3), dominant *Plantago maritima* stand on pebbly beach (island of Hiiumaa, PM4), flood-prone stony beach (Pärnu County, PM5).

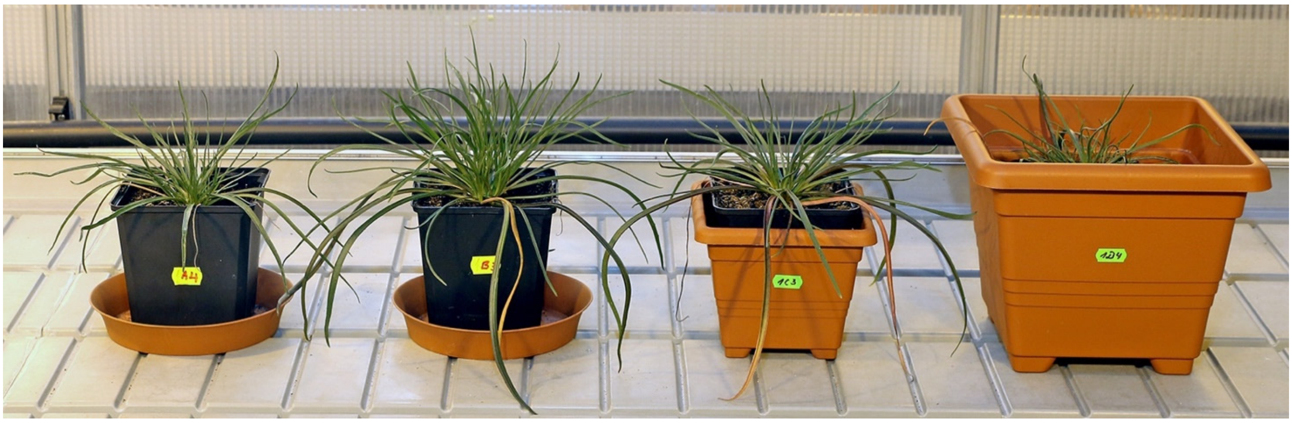

Figure S2. Typical *Plantago maritima* PM1 plants six weeks after the start of the treatment (two weeks after the flooding treatment was terminated). From left: 25% soil moisture, 50% soil moisture, 75% soil moisture, 80% F soil moisture.

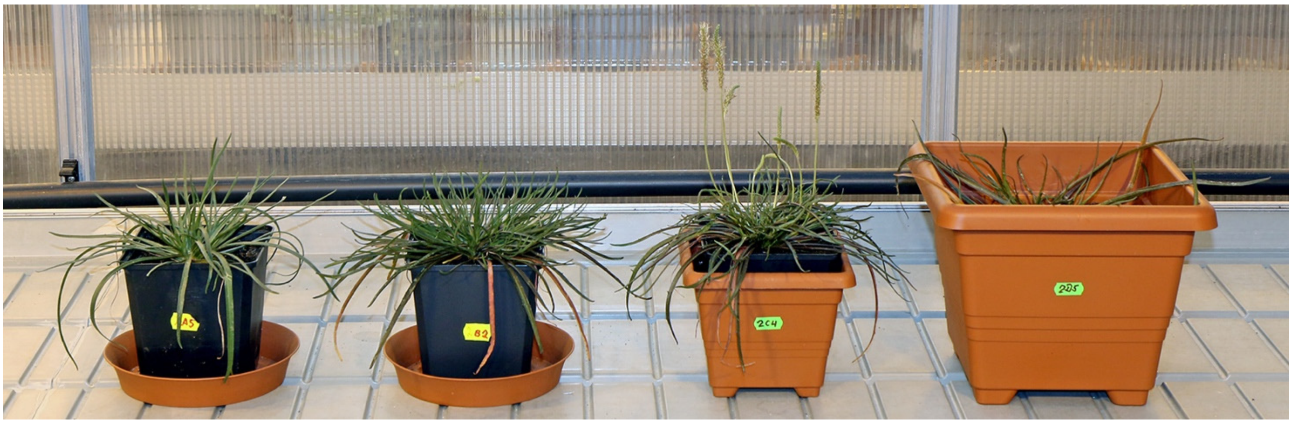

Figure S3. Typical *Plantago maritima* PM2 plants six weeks after the start of the treatment (two weeks after the flooding treatment was terminated). From left: 25% soil moisture, 50% soil moisture, 75% soil moisture, 80% F soil moisture.

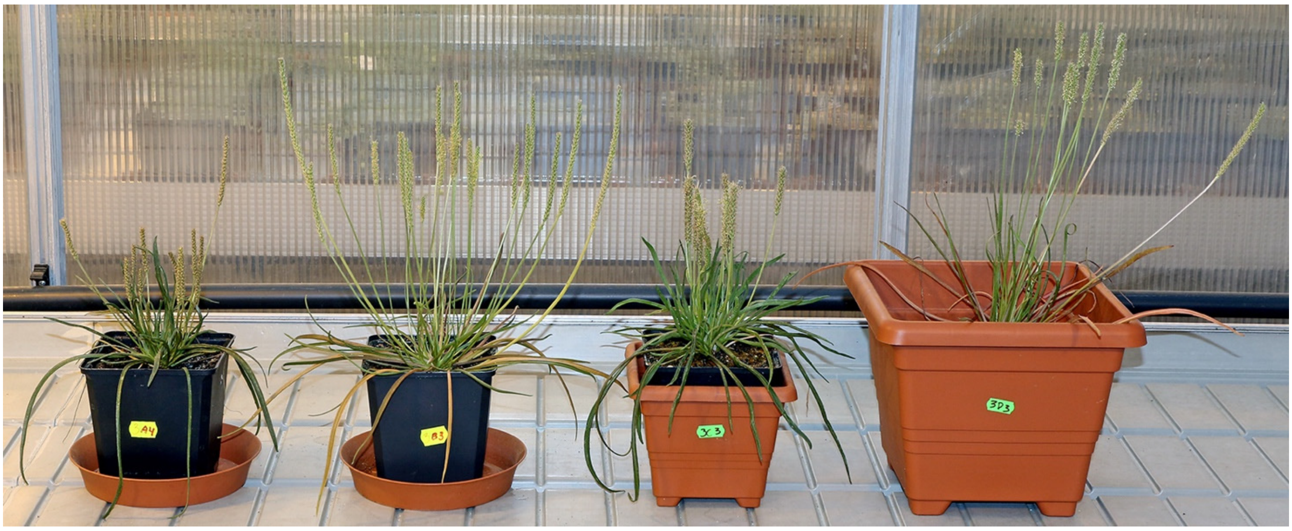

Figure S4. Typical *Plantago maritima* PM3 plants six weeks after the start of the treatment (two weeks after the flooding treatment was terminated). From left: 25% soil moisture, 50% soil moisture, 75% soil moisture, 80% F soil moisture.

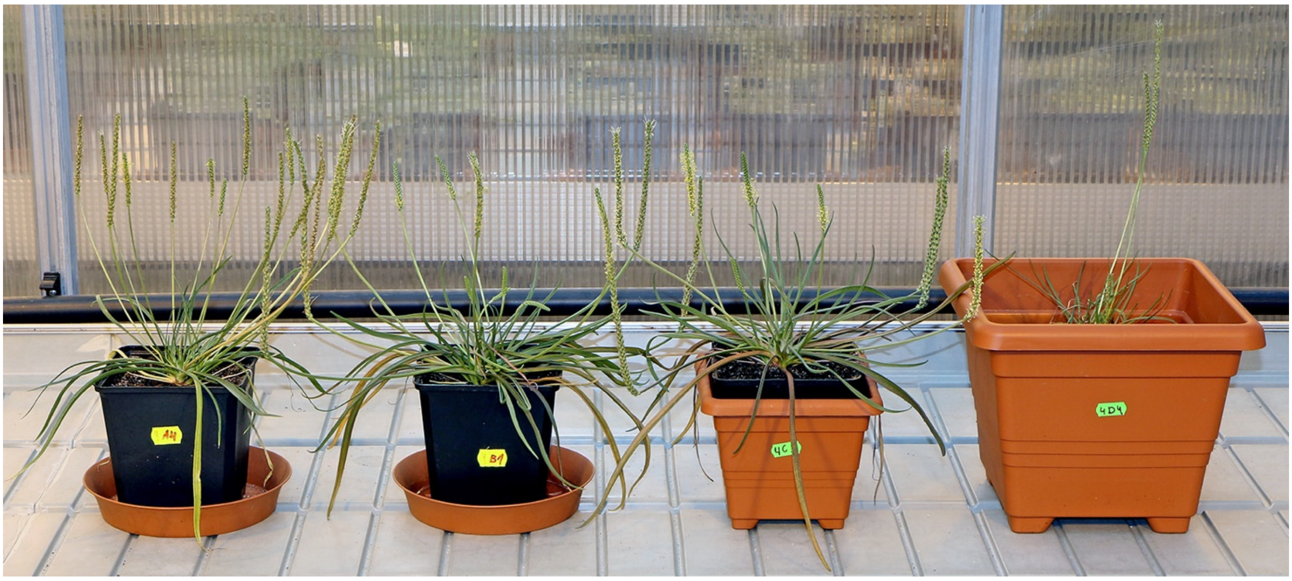

Figure S5. Typical *Plantago maritima* PM4 plants six weeks after the start of the treatment (two weeks after the flooding treatment was terminated). From left: 25% soil moisture, 50% soil moisture, 75% soil moisture, 80% F soil moisture.

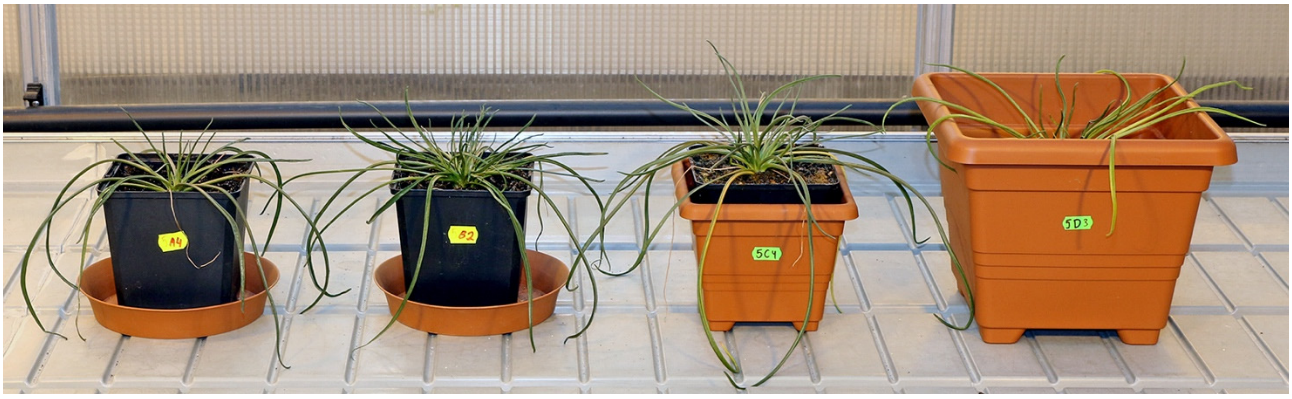

Figure S6. Typical *Plantago maritima* PM5 plants six weeks after the start of the treatment (two weeks after the flooding treatment was terminated). From left: 25% soil moisture, 50% soil moisture, 75% soil moisture, 80% F soil moisture.
