# Supplementary material for: Comparison of Growth and Physiological Effects of Soil Moisture Regime on Plantago maritima Plants from Geographically Isolated Sites on the Eastern Coast of the Baltic Sea
Source: Plants (Basel). 2024 Feb 25;13(5):633. doi: 10.3390/plants13050633 (PMC10934305; doi:10.3390/plants13050633)
Supplement: Supplementary file 1 [file plants-13-00633-s001.zip › Table Suppl.pdf]

Comparison of Growth and Physiological Effects of Soil Moisture Regime on *Plantago maritima* Plants from Geographically Isolated Sites on the Eastern Coast of the Baltic Sea

Katrīna Anna Ozoliņa, Astra Jēkabsone, Una Andersone-Ozola and Gederts Ievinsh

Table S1. Effect of soil moisture and flooding (F) on electrical conductivity, Na^+ concentration, K^+ concentration, and osmotic activity in roots of *Plantago maritima* plants from different accessions. Data are means ± SE from five replicates. Different letters indicate statistically significant differences according to the Tukey HSD test ($p < 0.05$) for the particular parameter. DM, dry mass.

Moisture	PM1	PM2	PM3	PM4	PM5
Electrical conductivity (mS m^{-1} kg^{-1} DM)					
25%	44.6 ± 1.0 abcd	44.6 ± 4.4 abcd	37.1 ± 2.8 bcd	45.8 ± 2.4 abcd	49.6 ± 1.6 abc
50%	40.2 ± 7.0 abcd	29.5 ± 1.3 d	33.7 ± 1.1 cd	50.4 ± 3.4 abc	45.2 ± 1.5 abcd
75%	56.3 ± 6.6 a	54.5 ± 1.8 ab	54.4 ± 3.4 b	48.8 ± 3.7 abc	52.8 ± 5.1 ab
80% F	42.9 ± 4.1 abcd	38.1 ± 0.9 bcd	51.2 ± 4.5 abc	44.8 ± 1.7 abcd	50.3 ± 2.9 abc
Na^+ concentration (g kg^{-1} DM)					
25%	2.08 ± 0.10 bc	1.45 ± 0.21 c	1.32 ± 0.18 c	1.83 ± 0.18 bc	2.72 ± 0.30 b
50%	1.73 ± 0.12 bc	1.61 ± 0.24 c	1.06 ± 0.08 c	3.89 ± 0.49 a	1.49 ± 0.11 c
75%	2.00 ± 0.13 bc	2.73 ± 0.23 b	1.90 ± 0.16 bc	1.83 ± 0.08 bc	1.57 ± 0.10 c
80% F	2.08 ± 0.14 bc	1.48 ± 0.10 c	1.83 ± 0.12 bc	1.88 ± 0.33 bc	1.88 ± 0.29 bc
K^+ concentration (g kg^{-1} DM)					
25%	12.1 ± 0.3 defg	15.8 ± 1.6 bcdefg	11.8 ± 1.0 efg	18.2 ± 1.1 abcdef	20.1 ± 0.6 abcd
50%	10.7 ± 2.5 fg	7.7 ± 0.6 g	12.1 ± 0.6 defg	16.8 ± 2.1 abcdef	20.7 ± 0.6 abc
75%	16.8 ± 1.9 abcdef	18.8 ± 0.9 abcdef	20.8 ± 1.9 abc	19.8 ± 2.3 abcde	22.3 ± 3.0 ab
80% F	11.2 ± 1.5 fg	13.7 ± 0.4 cdefg	20.3 ± 2.3 abc	19.4 ± 1.2 abcde	23.9 ± 1.4 a
Osmotic activity (osmol kg^{-1} DM)					
25%	1.23 ± 0.09 def	1.46 ± 0.05 bcde	1.47 ± 0.07 bcde	1.50 ± 0.14 bcde	1.44 ± 0.06 bcde
50%	0.90 ± 0.06 f	1.19 ± 0.16 ef	1.52 ± 0.11 abcde	1.28 ± 0.02 cdef	1.36 ± 0.06 bcde
75%	1.65 ± 0.13 abcd	1.71 ± 0.02 abc	1.58 ± 0.13 abcde	1.45 ± 0.06 bcde	1.78 ± 0.02 ab
80% F	1.38 ± 0.06 bcde	1.52 ± 0.03 bcde	1.91 ± 0.08 a	1.74 ± 0.06 ab	1.90 ± 0.07 a
Non-ionic osmotic activity (osmol kg^{-1} DM)					
25%	0.21 ± 0.09 abcd	0.31 ± 0.05 abcd	0.51 ± 0.04 ab	0.26 ± 0.11 abcd	0.08 ± 0.03 cd
50%	0.05 ± 0.03 cd	0.41 ± 0.15 ab	0.57 ± 0.09 a	0.05 ± 0.03 cd	0.02 ± 0.02 d
75%	0.41 ± 0.11 abc	0.31 ± 0.06 abcd	0.15 ± 0.05 bcd	0.16 ± 0.06 bcd	0.37 ± 0.10 abcd
80% F	0.40 ± 0.03 abc	0.46 ± 0.03 ab	0.51 ± 0.05 ab	0.38 ± 0.07 abcd	0.32 ± 0.03 abcd
